# Predicting metabolite-disease associations based on KATZ model

**DOI:** 10.1186/s13040-019-0206-z

**Published:** 2019-10-26

**Authors:** Xiujuan Lei, Cheng Zhang

**Affiliations:** School of Computer Science, Shaanxi Normal University, Xi’an, 710119 Shaanxi China

**Keywords:** Metabolite-disease associations, Heterogeneous network, KATZ

## Abstract

**Background:**

Increasing numbers of evidences have illuminated that metabolites can respond to pathological changes. However, identifying the diseases-related metabolites is a magnificent challenge in the field of biology and medicine. Traditional medical equipment not only has the limitation of its accuracy but also is expensive and time-consuming. Therefore, it’s necessary to take advantage of computational methods for predicting potential associations between metabolites and diseases.

**Results:**

In this study, we develop a computational method based on KATZ algorithm to predict metabolite-disease associations (KATZMDA). Firstly, we extract data about metabolite-disease pairs from the latest version of HMDB database for the materials of prediction. Then we take advantage of disease semantic similarity and the improved disease Gaussian Interaction Profile (GIP) kernel similarity to obtain more reliable disease similarity and enhance the predictive performance of our proposed computational method. Simultaneously, KATZ algorithm is applied in the domains of metabolomics for the first time.

**Conclusions:**

According to three kinds of cross validations and case studies of three common diseases, KATZMDA is worth serving as an impactful measuring tool for predicting the potential associations between metabolites and diseases.

## Background

Metabolism, a generic term for a series of ordered chemical reactions, plays a critical role in maintaining human life such as the growth and reproduction of organisms and the reaction to the external environment in body [1–3]. Numerous researches and experiments have indicated that some kinds of metabolites in concentration are distinct when people get ill compared with healthy people [4]. Hence, relevant metabolite-disease association is one of the significant judgements for doctors to diagnosing and treatment [4]. There are many examples such as diabetes. When it comes to blood sugar, people maybe think of one disease named diabetes naturally. Because the concentration of blood sugar in diabetes patient’s body is usually higher than normal body. In the past 10 years, Many metabolites which changed significantly such as the concentration of blood sugar have been gradually known as one of the criteria for doctors to diagnose diabetes after a quantity of experiments and clinical cases [5]. Based on the above example, it apparently reveals that metabolites also play an indispensable role in researching human diseases, which increasingly become a hot topic to explore the associations of them.

With the improvement of high-throughput metabolomics technologies, researchers could obtain a great deal of precious information. Meanwhile, metabolomic databases have been gradually developed, which is critical to the development of metabolomics [6]. For instance, HMDB database [7] which contains reliable information of human metabolites has continued to grow and evolve with enhancement and expansion of existing data from version 1.0 to 4.0 [7]. However, the identification of the associations between metabolites and diseases is only a tip of the iceberg, which indicates that thousands of potential metabolism and disease associations need to be tested and proved. However, conventional biology experiments can be tested and verified some assumptions but usually take a considerable time to get results. If the bias of results and assumptions are too large or results are not much more significant, experimenters may have to bear the financial loss. Thus, it is more important to develop computational methods which can save experimental time and fund and supply available prediction results. Some relevant methods of predicting potential associations between different biological molecules have been delivered for genomics such as gene-disease correlations [8–10], transcriptomics like circRNA-disease associations [11, 12] and proteomics such as identification of essential proteins [13–15], but the computational methods for predicting metabolite-disease associations are very few such as “Identifying diseases-related metabolites using random walk” [16] which is the first method to explore the latent associations and promote the development of computational method in metabolomics. However, they only consider the disease similarity when calculating metabolite similarity. In order to make full use of the known data, we use metabolite GIP kernel similarity to metabolite similarity and add the integrated disease similarity to calculate the predicted results.

In this study, we put forward one computational method named KATZMDA to explore novel metabolite-disease associations. Our proposed method is enlightened by KATZ algorithm, which has been utilized to predict the associations in social networks. Our computational method mainly consists of three steps: Firstly, the raw resources which come from the newest version of HMDB are gained for the basic data of prediction. Secondly, we compute the similarity for metabolites and diseases to rich types of data, where metabolite similarity network is computed by metabolite GIP kernel similarity while the improved disease GIP kernel similarity sub-network and semantic similarity sub-network are integrated into the disease similarity network. Thirdly, we predict metabolite-disease associations based on KATZ algorithm. Finally, we adopt the leave-one-out cross validation (LOOCV) and 5-fold and 10-fold cross validation to evaluate the performance of KATZMDA which acquired the AUC (area under the ROC curve) values of 0.9186, 0.8897+/− 0.0173 and 0.9029+/− 0.0073, respectively. For the sake of further verification, we utilize case studies of Liver disease, Cerebral infarction and Gestational diabetes, respectively. What’s more, the values of AUC confirm that our method is better than other methods in section of Comparison with other methods. Therefore, the results indicate that KATZMDA is forceful and dependable in predicting potential metabolite-disease associations.

## Results

### Leave-one-out cross validation (LOOCV)

**Fig. 1:**
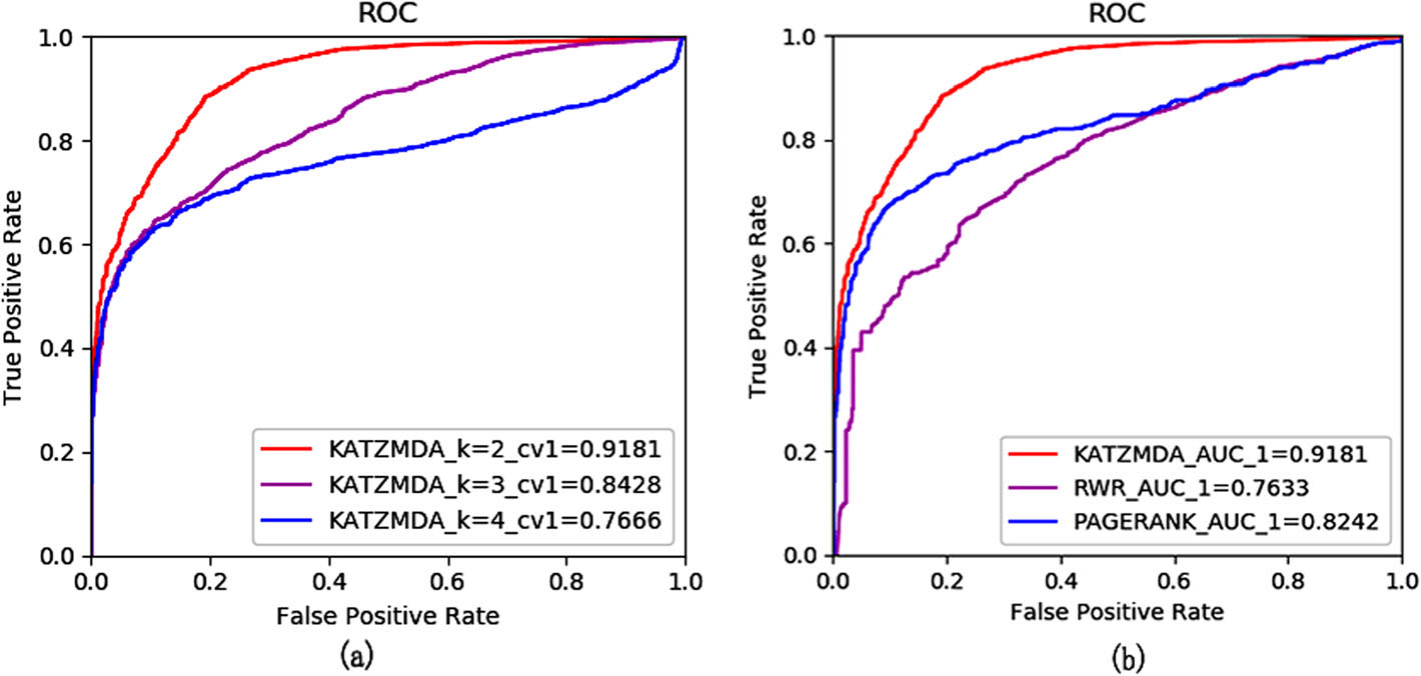
The ROC about LOOCV. **a** The AUCs of KATZMDA when k = 2,3,4 for LOOCV **b** Comparison of KATZMDA with other computational methods for LOOCV

It is a common tool for LOOCV to evaluate the performance of our proposed computational method. In LOOCV, if one known association of metabolite and disease is used as a test set, the rest of known associations are regarded as training sets and the unknown associations become as candidate sets. Finally, a result will be obtained when all the known associations take turns as test sets. There are 4537 known metabolite-disease associations, so our experiment needs to be run 4538 times. In every loop, the test sample is considered as successful prediction result if the rank of the test sample is beyond the given threshold. According to changing thresholds, we finally acquire a series of values about True Positive Rate (*TPR*, sensitivity) and False Positive Rate (*FPR*, 1-specificity), which can help to depict the ROC curve. The prediction performance in our model is gained after calculating AUC. If the AUC tends to 1, the performance will be perfect. Moreover, when the AUC tends to 0.5, it indicates that the performance is random. If the AUC tends to 0, the performance is terrible. With several experiments, we find that our proposed computational model acquires better LOOCV performance that the relevant AUC value is 0.90 when parameter *k* is equal to 2. While, if parameter *k* is beyond to 2, the AUC will drop down (see Fig. 1 (a)).

### K-fold cross validation

**Fig. 2:**
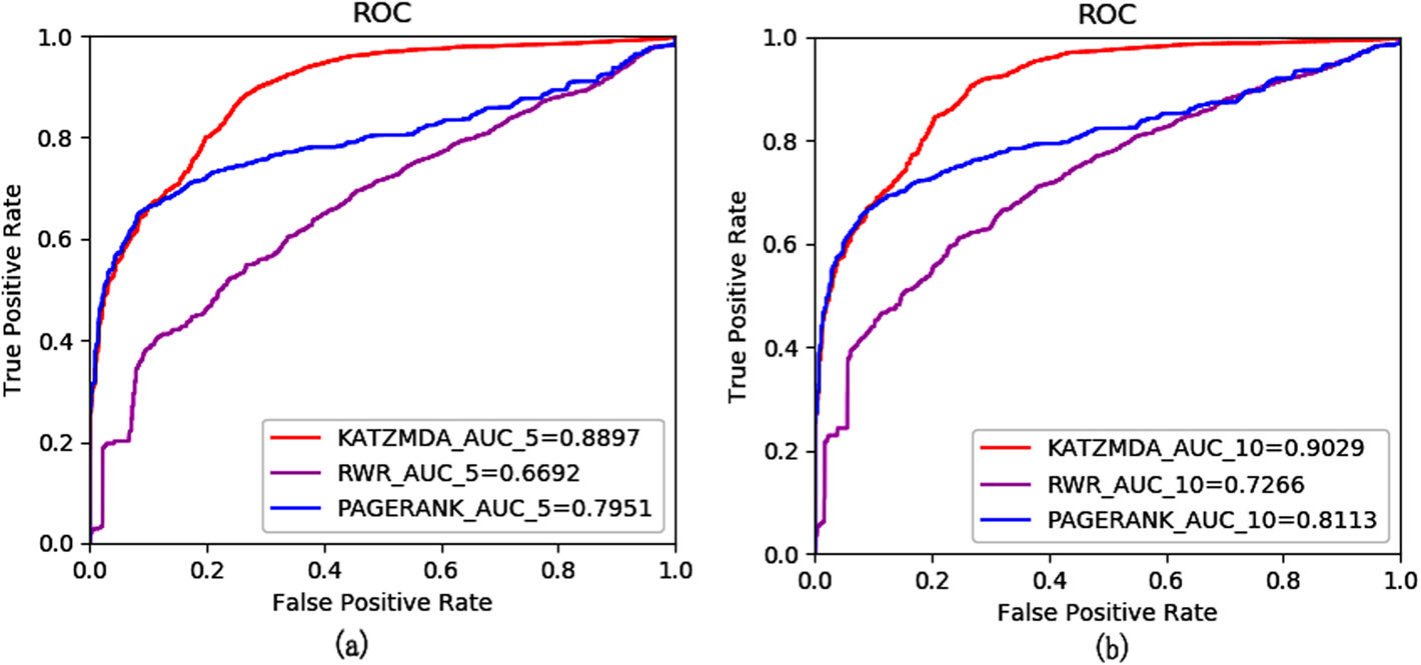
The ROC about k-fold cross-validation. Comparison of KATZMDA with other computational methods for (**a**)5-fold cross-validation, (**b**)10-fold cross-validation

K-fold cross validation is also implemented for the performance evaluation of our method. In K-fold cross validation, all the known metabolite-disease pairs are randomly and averagely decomposed *k* parts. One part is regarded as a test sample, then the rest of parts (*k*-1) is utilized for training. As above mentioned in LOOCV, unknown relations in metabolite-disease pairs are utilized as candidate samples in K-fold cross validation. Specifically, 5-fold and 10-fold cross validation are adopted to deeply evaluate the prediction performance of KATZMDA. Given the influence of the latent bias, when dividing random sets for evaluating performance, we set this experiment to loop many times, then the correlative ROC curves and AUCs are acquired as LOOCV. Lastly, we get the one of AUCs’ group of these two types of cross validation which are 0.8897 and 0.9029, respectively (see Fig. 2).

### Comparison with other methods

In order to evaluate the performance of KATZMDA in predicting potential metabolite-disease associations, we compare KATZMDA with the methods such as random walk restart (RWR) and PageRank method and implement the validation experiments mentioned above on each method based on the same dataset. In RWR, we use the same parameters as Hu’s method [16]. For LOOCV, RWR, PageRank gained AUCs of 0.7633,0.8242, respectively. For 5-fold cross validation, RWR, PageRank gained AUCs of 0.6692, 0.7951, respectively. For10-fold cross validation, RWR, PageRank gained AUCs of 0.7266, 0.8113, respectively (see Fig. 2). According to these evaluation mechanisms, KATZMDA can obtain higher AUC value. It means that KATZMDA is more effective than those compared methods and has a latent capability to explore more novel metabolite-disease associations.

### Parameters analyzing

**Fig. 3:**
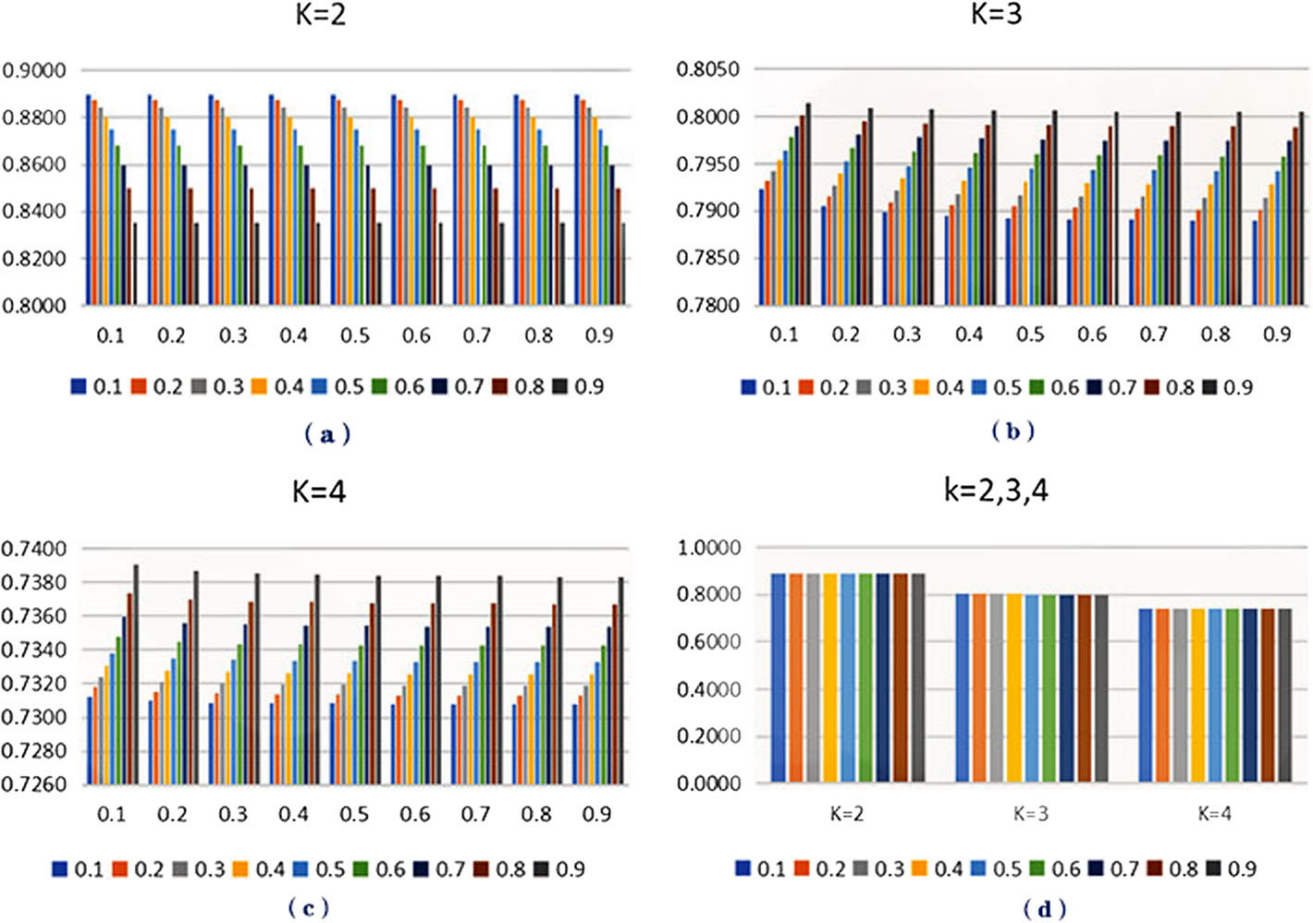
Parameter analysis. **a, b**, **c**: x-axis represents *δ*, y-axis represents AUC, different colors represent different values of γ. **d**: when k = 2, γ = 0.1, δ = [0.1,0.9];when k = 3, γ = 0.9, δ = [0.1,0.9]; when k = 4, γ = 0.9, δ = [0.1,0.9]; different colors represent different values of *δ*

In this section, we are committed to find the influence of some parameters and the best parameters on our proposed method. Then we analyze the following parameters: *γ* as a weighted parameter determines the proportion of the two types of disease similarities which affects the final disease similarity. So, it is essential to analyze it which is changed from 0.1 to 0.9 (see Table 1). Referring to the previous study, the parameter *δ* is selected below 1/‖ *M* ‖ ^2^. However, we change its value as *γ* to explore its effect to our method (see Table 2). We find that it is steadier for AUC when changing *δ* and then we set 0.1 to the best value. The parameter *k* which represents the length of path between metabolites and diseases is always set 3 but we find the suitable value of *k* is 2 when obtaining the best estimated performance after several tests in our experiment (see Tables 1 and 2, Fig. 1 (a)). The results of different values of *k* are displayed (see Tables 1 and 2, Fig. 3 (a-c)). Considering the efficiency of time, we adopt the five-fold cross validation to calculate above results. Finally, we select the best parameters group in each value of *k* for comparison (see Fig. 3 (d)). The best parameters are set as follows: *k* = 2, *γ* =0.1 and *δ* =0.1, respectively.

**Table 1:** The AUC values based on changing *γ* and *k* (*δ =*0.1)

	γ=0.1	γ=0.2	γ=0.3	γ=0.4	γ=0.5	γ=0.6	γ=0.7	γ=0.8	γ=0.9
K = 2	0.8897	0.8874	0.8842	0.8800	0.8747	0.8681	0.8600	0.8496	0.8351
K = 3	0.7923	0.7932	0.7942	0.7953	0.7965	0.7977	0.7989	0.8002	0.8014
K = 4	0.7313	0.7318	0.7324	0.7330	0.7338	0.7348	0.7360	0.7374	0.7391

**Table 2:** The AUC values based on changing *δ* and *k* (*γ* = 0.1)

	δ=0.1	δ=0.2	δ=0.3	δ=0.4	δ=0.5	δ=0.6	δ=0.7	δ=0.8	δ=0.9
K = 2	0.8897	0.8897	0.8897	0.8897	0.8897	0.8897	0.8897	0.8897	0.8897
K = 3	0.7923	0.7904	0.7898	0.7894	0.7892	0.7891	0.7890	0.7889	0.7889
K = 4	0.7401	0.7319	0.7313	0.7310	0.7309	0.7308	0.7308	0.7308	0.7308

### Case study

**Fig. 4:**
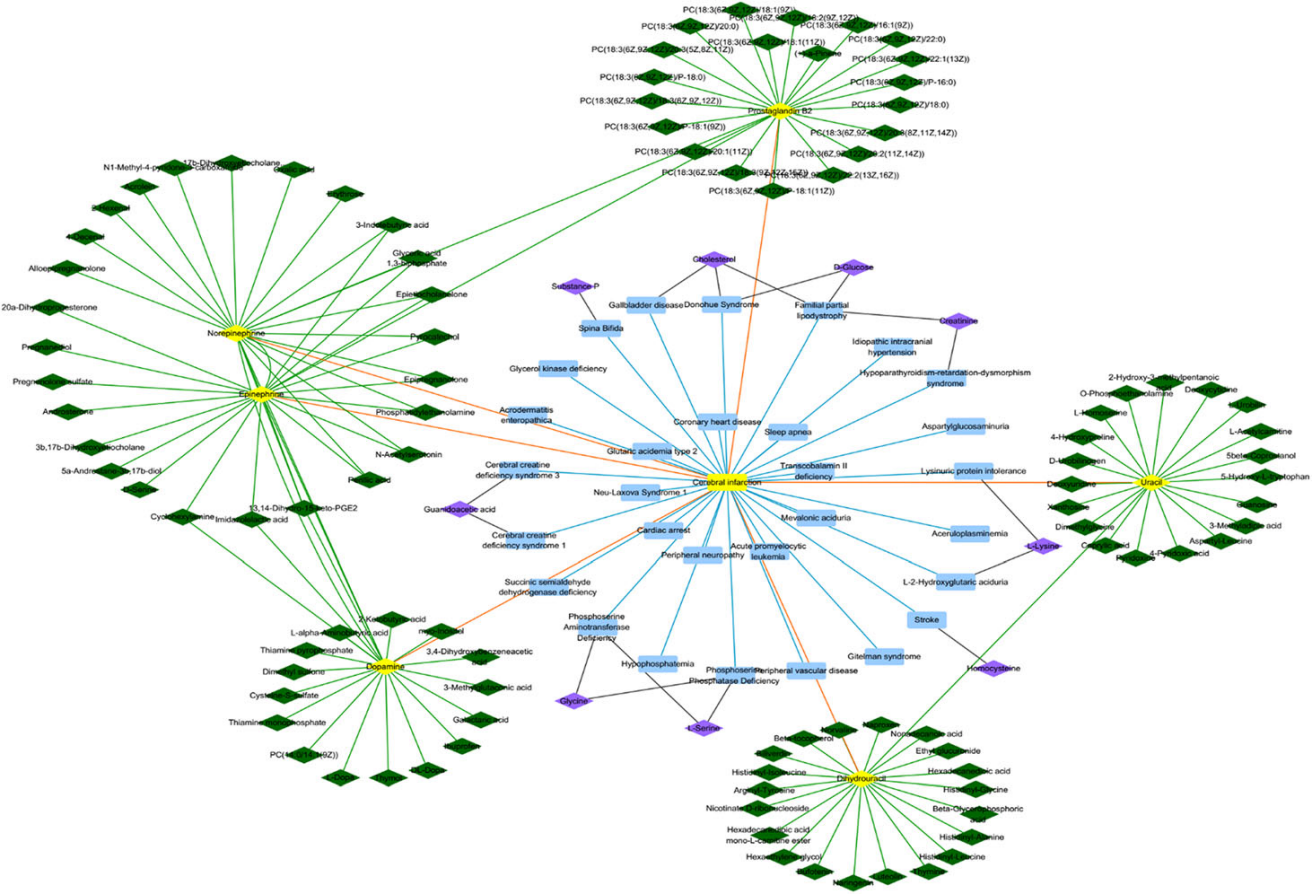
The network between the prediction of metabolites and two kinds of neighbors. This graph shows which of these two kinds of neighbors have more contributions to the prediction of metabolites. Rectangle represents the diseases. Green color represents the neighbors of known metabolites about Cerebral infarction whose ranks of similarity are top 20 and the associations between them. Yellow color represents Cerebral infarction and its relevant metabolites. Blue color represents the neighbors of Cerebral infarction and their relations whose similarity scores are above 0.6. Purple represents the predicted metabolites about Cerebral infarction and the black edges represent the links between the neighbors of Cerebral infarction and the predicted metabolites about Cerebral infarction

In this section, we have taken several diseases as examples to make case studies, which can make us deeply realize the associations between metabolites and diseases. There are three common diseases which are Liver disease, Cerebral infarction and Gestational diabetes, respectively. Considering the accuracy of results in our method, we find some details in published papers to prove the relevant prediction associations. For the above mentioned diseases, we select the neighbors of themselves and their relevant known metabolites to seek the associations between these two types of neighbors and predictive metabolites, respectively, which takes Cerebral infarction as an example showing in Fig. 4.

Liver disease means a lesion that occurs in the liver and happens all the time around people. It is a total name of high-risk disease about liver, which includes viral hepatitis, liver abscess, alcoholic hepatitis and fatty liver. We carry out a case study of liver disease with our method. Finally, there are top 10 predicted metabolites having been confirmed to have some influence on the liver disease patients by calculating known associations on our method (see Table 3). Taking follows as examples, Glycine(1st) is proved to not only treat alcoholic hepatitis, but also prevent and treat hepatocellular carcinoma in alcoholic cirrhosis [17]. What’s more, Glycine [18] is a kind of effect immuno-nutrient substance when treated diverse chronic liver diseases [17]. L-Serine, Creatine, L-Tryptophan, Cholesterol (2nd, 3rd, 4th, 9th) were revealed to have significant influence to one kind of Liver disease named fatty liver [19–22].

**Table 3:** Candidate metabolites of liver disease

Liver disease		
Rank	Metabolite name	Evidences
1	Glycine	PMID: 16344603
2	L-Serine	PMID: 25644346
3	Creatine	PMID: 26832170
4	Cholesterol	PMID: 28733574
5	L-Alanine	PMID: 1742521
6	L-Lysine	PMID: 7890898
7	L-Phenylalanine	PMID: 17615399
8	L-Tyrosine	PMID: 22847184
9	L-Tryptophan	PMID: 21841000
10	Creatinine	PMID: 26311594

Cerebral infarction is one of the most common diseases in cerebrovascular disease. In the Cerebral infarction-related metabolites prediction results, top 10 predicted metabolites have been verified. by published references (see Table 4). For instance, Glycine could abate Cerebral infarction caused by ischemia/reperfusion in mice [23].

**Table 4:** Candidate metabolites of Cerebral infarction

Cerebral infarction		
Rank	Metabolite name	Evidences
1	Glycine	PMID: 22796215
2	L-Serine	PMID: 20476571
3	Cholesterol	PMID: 26957269
4	Homocysteine	PMID: 27079234
5	Creatine	PMID: 24396424
6	Creatinine	PMID: 28326034
7	L-Lysine	PMID: 28900508
8	Guanidoacetic acid	PMID: 27497517
9	Substance P	PMID: 27338372
10	D-Glucose	PMID: 23428707

Gestational diabetes is one kind of common diseases which affects 5 to 6% of pregnant women [24]. There are some predicting associations which shows top 10 predicted metabolites and 9 of top 10 predicted Gestational diabetes-related metabolites have been certified (see Table 5). More and more details indicated that the Substance might be a new role which lead not only to the development of diabetes gestational diabetes, but also diabetes mellitus type 2 [24]. Although there is no clear evidence to confirm the associations between Guanidoacetic acid and Gestational diabetes, some experimental literatures show that the detection of Guanidoacetic acid is an available indicator for renal tubular dysfunction in the early phase of diabetes mellitus [25].

**Table 5:** Candidate metabolites of Gestational diabetes

Gestational diabetes		
Rank	Metabolite name	Evidences
1	Glycine	PMID: 28278310
2	Cholesterol	PMID: 29778664
3	L-Serine	PMID: 26406294
4	Creatinine	PMID: 29728364
5	L-Lysine	PMID: 25419905
6	Creatine	PMID: 25925942
7	Substance P	PMID: 24720596
8	Homocysteine	PMID: 27180921
9	Guanidoacetic acid	Unconfirmed
10	D-Glucose	PMID: 10855532

## Discussions

Large quantities of evidences have revealed that metabolites in human body are implicated in reflecting human physiological such as complicated disease pathology. Although biotic experiments can explore potential metabolite-disease associations and help people acquire data which we need. However, these methods are time-consuming and expensive. Here, we put forward a practical method named KATZMDA, which not only guarantees the accuracy of predicting the latent associations between metabolites and diseases but also effectively cuts down the time and investment. In this study, we firstly calculate metabolite/disease similarities by combining their relevant similarities. Secondly, we establish a heterogeneous network based on metabolites-disease associations network, metabolites similarity network and diseases similarity network. According to different paths with different lengths, KATZMDA searches on a heterogeneous network and computes a final score for each pair of metabolite and disease which could estimate whether the disease has association with the metabolite or not.

Experimental results testify the superior performance of KATZMDA compared with other methods in this study. There are some advantages as follows. Firstly, considering the characteristic of data, KATZ algorithm is applied in predicting associations of metabolites and diseases, which lays a foundation for the effectiveness of our final predictions. Secondly, we add properties of topology and biology in disease similarity networks. Simultaneously, we set an adaptive parameter to balance the two kind of properties in order to better explore the potential relationships.

Although better prediction results are obtained by KATZMDA, some limitations still can’t be neglected. For the original data, the associations proved between metabolites and diseases in the domain of metabolomic are far from satisfied. Additionally, it is out-off-balance between the proportion of positive samples and negative samples because of the sparse data. So only one thing we can do is trying to reduce the number of negative samples to the same number of positive samples by randomly selecting negative samples. What’s more, the similarity of metabolite-metabolite pairs, one of significant factor to guarantee the accuracy of result in theory, only has few contributions to the prediction (see Fig. 4). Therefore, we need to take their biological characteristics besides topological characteristics into consideration in the future.

## Conclusions

According to mining a great deal of useful resources about metabolites and diseases, we can get reliable prediction scores to generate new hypotheses between metabolites and diseases by our methods, which may be of benefit to identify new research trends and boost interdisciplinary studies. The experimental results indicated our method is powerful. Moreover, three common diseases are used to be analyzed which deeply demonstrates applicability of the method. Uncovering metabolite-disease associations are of great significance in understanding disease mechanism’s and advancing biology through integrated interdisciplinary research.

## Methods

### Human metabolite-disease associations network

**Fig. 5:**
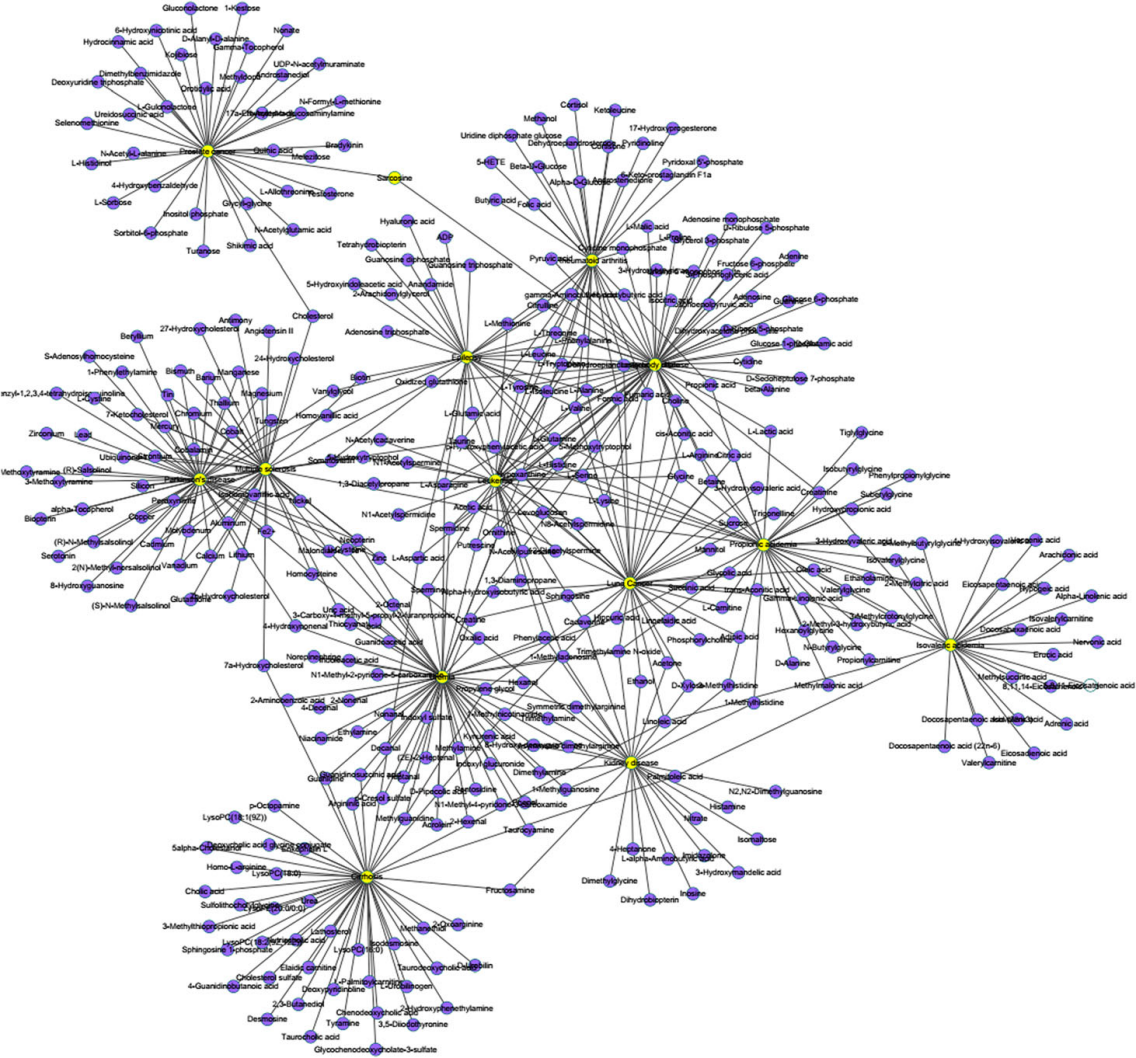
A part of the known metabolite-disease associations network. Yellow nodes represent diseases and purple nodes represent metabolites

The known metabolite-diseases associations are extracted from the Human Metabolome Database(HMDB) which has abundant information about small molecule metabolites found in the human body [7]. In this study, we download the data about HMDB and extract the associations between metabolites and diseases. Considering that we need to use disease semantic similarity in our method, then we select the diseases with DOID and its relevant metabolites from the associations which has been extracted. Finally, 4537 metabolite-diseases associations are extracted from the initial data, which consist of 216 diseases and 2262 metabolites to be established the known metabolite-disease associations network(see Fig. 5). For the sake of simplicity of expression, an adjacency matrix *M*(nd*nm) is constructed to describe metabolite-disease associations, where *nm* and *nd* represent the number of metabolites and diseases, respectively. If a disease *i* has been approved to have an association with a metabolite *j*, then *M*(*i,j*) = 1, otherwise, *M*(*i,j*) = 0.

### Disease semantic similarity

According to the Mesh Database, we can obtain some detailed information about diseases because every disease has their own unique DAG (Directed Acyclic Graph) which reflects the correlations between diseases [26]. As an example of DAG about disease *D*, it could be defined as DAG(*D*) = (*D*, T(*D*), E(*D*)), where T(*D*) is composed by disease *D* itself and all its ancestor diseases and E(*D*) is composed by direct edges from a more general term (parent node) to a more specific term (child node). Additionally, the semantic value of disease *D* could be calculated as follows [26, 27]:

1 $$ {D}_V(D)=\sum \limits_{d\in T(D)}{D}_D(d) $$

2 $$ {D}_D(d)=\left\{\begin{array}{c}\ 1\ \\ {}\mathit{\max}\left\{\Delta  \ast {D}_D\left({d}^{\prime}\right)|{d}^{\prime}\in ch\mathrm{i} ldren\ of\ d\ \right\}\ \end{array}\right.{\displaystyle \begin{array}{c} if\ d=D\\ {} if\ d\ne D\end{array}} $$

where ∆ is a factor affecting the semantic contribution of connecting parent node *d* with its child node *d’*. For a given disease *D*, there are negative correlations that the nodes far from disease *D* have less semantic contribution to *D*. Moreover, there are same semantic contributions to disease *D* between nodes whose positions are at the same levels [26]. Finally, *DSS* is used to represent disease semantic similarity matrix. The semantic similarity between disease *i* and *j* could be calculated as follows:

3 $$ DSS\left(d(i),d(j)\right)=\frac{\sum_{t\in T\left(D(i)\right)\cap T\left(D(j)\right)}\left(D(i)(t)+D(j)(t)\right)}{DV\left(D(i)\right)+ DV\left(D(j)\right)} $$

### GIP kernel similarity

GIP kernel similarity is applied in the association network of biological information nodes to measure similarity based on their topological structures [28]. According to the metabolite-disease associations network and the hypothesis that similar metabolites are more likely to reflect a similar pattern of interaction and non-interaction with diseases, GIP kernel similarity of metabolites could calculated as follows [29]:

4 $$ GM\left(m(i),m(j)\right)=\mathit{\exp}\left(-{\omega}_m{\left\Vert IP\left(m(i)\right)- IP\left(m(j)\right)\right\Vert}^2\right) $$

where the interaction profile *IP*(*m*(*i*)) of metabolite *m*(*i*), a binary vector, can be gained according to whether a metabolite *m*(*i*) is associated with each disease. *ω*_*m*_ influences the kernel bandwidth, which is calculated as follows:

5 $$ {\omega}_m={\omega^{\hbox{'}}}_m/\left(\frac{1}{n_m}{\sum}_{k=1}^{n_m} IP{\left(m(i)\right)}^2\right) $$

where *n*_*m*_ represents the number of metabolites in metabolite and disease associations network. For simplifying experiment, *ω*_*m*_ is usually set as 1 according to previous research [28]. Thereby, metabolites GIP kernel similarity matrix (*GM*) is acquired. Then, we can get a metabolite similarity network (*MS*) based on the *GM* matrix*.* Similar as the way to set up metabolite similarity network, the disease similarity network (*DM*) is established by the disease GIP kernel similarity matrix(*GD*) which is computed as follows [29]:

6 $$ GD\left(d(i),d(j)\right)=\mathit{\exp}\left(-{\omega}_d{\left\Vert IP\left(d(i)\right)- IP\left(d(j)\right)\right\Vert}^2\right) $$

7 $$ {\omega}_d={\omega^{\hbox{'}}}_d/\left(\frac{1}{n_d}{\sum}_{i=1}^{n_d} IP{\left(d(i)\right)}^2\right) $$

According to the relevant research [30], it reveals that disease GIP kernel similarity which is transformed in logistic function enables to improve predictive accuracy. Hence, logistic function in the previous research is used [30] as follows:

8 $$ GD L\left(d(i),d(j)\right)=\frac{1}{1+{e}^{a\ast GD\left(d(i),d(j)\right)+b}} $$

where *a* = − 15, *b* = log(9999) [30]. *GDL* represents the improved disease GIP kernel similarity.

### Integrate similarity for diseases

In this part, in order to tackle the sparse data in disease semantic similarity matrix and improve the accuracy, a new similarity matric about disease (*SD*) is constructed which is composed by disease semantic similarity matrix *DSS* and improved disease GIP kernel similarity matrix (*GDL*). The computing formulas are as follows:

9 $$ SD\left(d(i),d(j)\right)=\left\{\begin{array}{c} GDL\left(d(i),d(j)\right)\ \\ {}\ \\ {}\left(1-\gamma \right) DSS\left(d(i),d(j)\right)+\gamma GD\left(d(i),d(j)\right)\end{array}\ \begin{array}{c} if\  DSS\left(i,j\right)=0\\ {}\ \\ {} otherwise\end{array}\ \right. $$

### KATZMDA

**Fig. 6:**
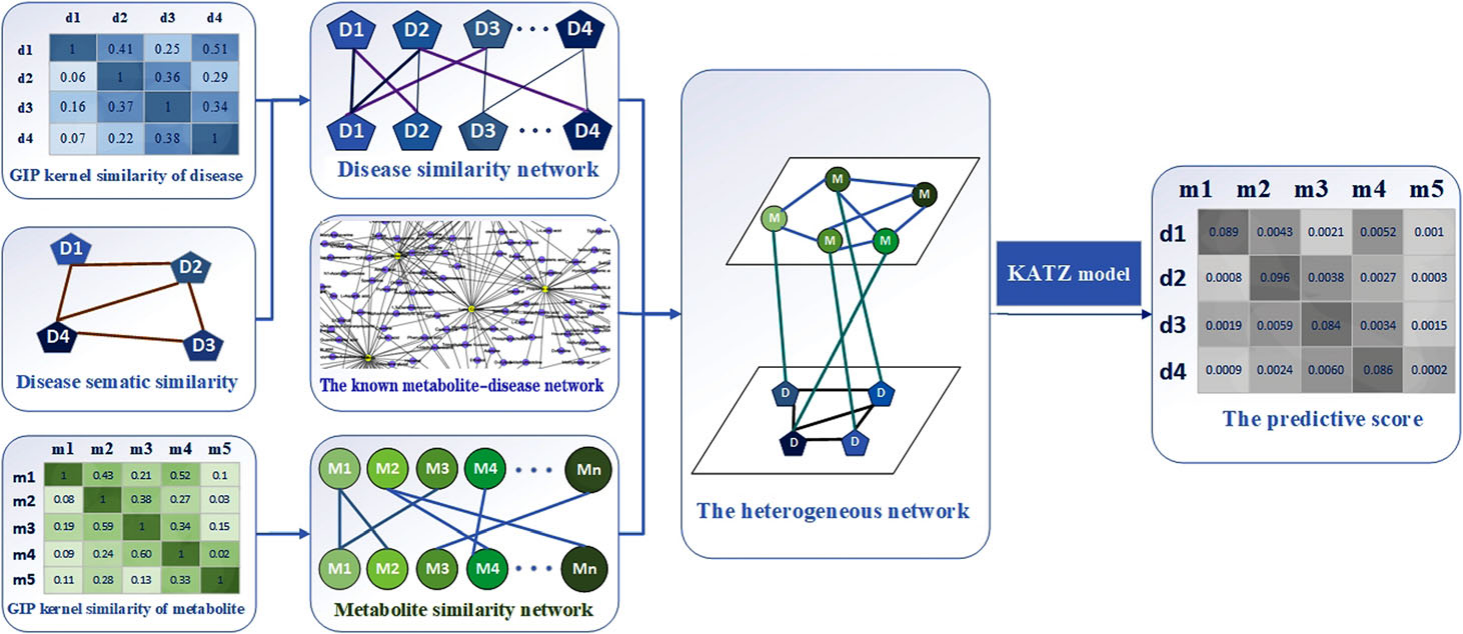
Flowchart of KATZMDA

KATZ, a set of methods to investigate the associations of society, has gradually spread in domains of bioinformatics. According to the number of paths between each two nodes and the length of each path, KATZ can calculate the score of each two nodes. The higher the score is obtained, the greater the potential correlation is. There are a great deal of experiments confirming its available performance such as identifying the latent associations of microbes and diseases, lncRNAs and environmental factors. Due to these successful experiences, the KATZMDA method has been adopted in predicting metabolite-disease associations in this study (see Fig. 6). This model in the heterogeneous network could obtain a score matrix which reflects the possible associations between each metabolite-disease pair. Generally, the paths’ number of metabolite *i*, disease *j* and the different length of different paths [31] needs to be taken into consideration, when we calculate the potential association between metabolite *i* and disease *j* in the known metabolite-disease associations network. M^*l^(*i,j*) represents the number of paths linking metabolite *i* and disease *j. k* represents the length of paths between metabolite *i* and disease *j*. Because of the existence of different length, we gather all paths with different lengths of metabolite *i* and disease *j*. According to the previous study [32, 33], it cannot be ignored that the longer paths have lower influence than shorter between each two nodes. So we adopt non-negative coefficient *δ* to control the influence of different-length paths [32]. If *l1 < l2,* then *δ 2< δ 1*. Accordingly, the latent associations of each metabolite-disease pair could be expressed as *Z*(*m*_*i*_*, d*_*j*_) of matrix *Z*:

10 $$ Z\left({m}_i,{d}_j\right)={\sum}_{l=1}^k{\delta}_l{M}^{\ast l}\left(i,j\right) $$

Gathering all associations between metabolite-disease pairs like the eq. (10):

11 $$ Z={\sum}_{l\ge 1}{\delta}^l{MD}^l={\left(I-\delta M\right)}^{-1}-I $$

where *Z* represents the similarity of all the metabolite-disease pairs. The parameter *δ* is chosen on the basis of *δ* < 1/||*M* ||^2^ in Zou’s method [33]. The adjacency matrix *M* is substituted by the following new form utilizing the similarity matrices of diseases and metabolites which were previously reconstructed as follows:

12 $$ {M}^{\ast }=\left[\begin{array}{cc} SM& M\\ {}{M}^T& SD\end{array}\right] $$

Additionally, when *k* is equal to 2, 3, 4, the calculation of the method can be showed as follows:

13 $$ {Z}^{k=2}\left({M}^{\ast}\right)=\delta \bullet M+{\delta}^2\bullet \left( SM\bullet M+M\bullet SD\right) $$

14 $$ {Z}^{k=3}\left({M}^{\ast}\right)={Z}^{k=2}\left({M}^{\ast}\right)+{\delta}^3\bullet \left(M\bullet {M}^T\bullet M+{SM}^2\bullet SD+ SM\bullet M\bullet SD+M\bullet {SD}^2\right) $$

15 $$ {Z}^{k=4}\left({M}^{\ast}\right)={Z}^{k=3}\left({M}^{\ast}\right)+{\delta}^4\bullet \left({SM}^3\bullet M+M\bullet {M}^T\bullet SM\bullet M+ SM\bullet M\bullet {M}^T\bullet M+\mathrm{M}\bullet SD\bullet {M}^T\bullet M\right)+{\delta}^4\bullet \left(M\bullet {M}^T\bullet M\bullet SD+{SM}^2\bullet M\bullet SD+ SM\bullet M\bullet {SD}^2+M\bullet {SD}^3\right) $$

## Data Availability

Please contact author for data requests.

## Abbreviations

AUC: Area under the curve
DAG: Directed Acyclic Graph
FPR: False positive rate
GIP: Gaussian interaction profile
LOOCV: Leave-one-out across validation
ROC: Receiver operating characteristic
TPR: True positive rate

## References

[1] Timofeeva Y, Lord GJ, Coombes SJNM (2011). Metabolite profiles and the risk of developing diabetes.

[2] Cheng L, Yang H, Zhao H, Pei X, Shi H, Sun J (2017). MetSigDis: a manually curated resource for the metabolic signatures of diseases.

[3] Lokhov PG, Maslov DL, Kharibin ON, Balashova EE, Archakov AI. Label-free data standardization for clinical metabolomics. BioData Mining. 2017;10(1):10. 10.1186/s13040-017-0132-x PMC5329969 28261328

[4] Huang W., Alexander G. E., Chang L., Shetty H. U., Krasuski J. S., Rapoport S. I., Schapiro M. B. (2001). Brain metabolite concentration and dementia severity in Alzheimer's disease: A 1H MRS study. Neurology.

[5] Lu Jingyi, Xie Guoxiang, Jia Weiping, Jia Wei (2013). Metabolomics in human type 2 diabetes research. Frontiers of Medicine.

[6] Hollywood Katherine, Brison Daniel R., Goodacre Royston (2006). Metabolomics: Current technologies and future trends. PROTEOMICS.

[7] Wishart David S, Feunang Yannick Djoumbou, Marcu Ana, Guo An Chi, Liang Kevin, Vázquez-Fresno Rosa, Sajed Tanvir, Johnson Daniel, Li Carin, Karu Naama, Sayeeda Zinat, Lo Elvis, Assempour Nazanin, Berjanskii Mark, Singhal Sandeep, Arndt David, Liang Yonjie, Badran Hasan, Grant Jason, Serra-Cayuela Arnau, Liu Yifeng, Mandal Rupa, Neveu Vanessa, Pon Allison, Knox Craig, Wilson Michael, Manach Claudine, Scalbert Augustin (2017). HMDB 4.0: the human metabolome database for 2018. Nucleic Acids Research.

[8] Zeng X, Ding N, Rodríguez-Patón A, Zou Q. Probability-based collaborative filtering model for predicting gene–disease associations. BMC Medical Genomics. 2017;10(5):76. 10.1186/s12920-017-0313-y PMC5751590 29297351

[9] Natarajan N., Dhillon I. S. (2014). Inductive matrix completion for predicting gene-disease associations. Bioinformatics.

[10] Lei X, Zhang YJIS. Predicting disease-genes based on network information loss and protein complexes in heterogeneous network. Information Sciences. 2018.

[11] Xiao Q, Luo J, Dai J. Computational prediction of human disease-associated circRNAs based on manifold regularization learning framework. IEEE Journal of Biomedical and Health Informatics. 2019;PP(99):1. 10.1109/JBHI.2019.2891779 30629521

[12] Yan C, Wang J, Wu F-X. DWNN-RLS: regularized least squares method for predicting circRNA-disease associations. BMC bioinformatics. 2018;19(19):520. 10.1186/s12859-018-2522-6 PMC6311892 30598076

[13] Lei X, Yang X, Wu F, Artificial fish swarm optimization based method to identify essential proteins. IEEE/ACM transactions on computational biology and bioinformatics. 2018. 10.1109/TCBB.2018.2865567 30113899

[14] Lei Xiujuan, Wang Siguo, Wu Fang-Xiang (2019). Identification of Essential Proteins Based on Improved HITS Algorithm. Genes.

[15] Lei X, Fang M, Wu FX, Chen L. Improved flower pollination algorithm for identifying essential proteins. BMC systems biology. 2018;12(4):46. 10.1186/s12918-018-0573-y PMC5998882 29745838

[16] Hu Y, Zhao T, Zhang N, Zang T, Zhang J, Cheng L. Identifying diseases-related metabolites using random walk. BMC bioinformatics. 2018;19(5):116. 10.1186/s12859-018-2098-1 PMC5907145 29671398

[17] Yamashina S, Ikejima K, Enomoto N, Takei Y, Sato NJAC, Research E. Glycine as a Therapeutic Immuno-Nutrient for Alcoholic Liver Disease. Alcoholism: Clinical and Experimental Research. 2005;29:162S–165S. 10.1097/01.alc.0000189281.82523.6c 16344603

[18] Luntz SP, Unnebrink K, Seibert-Grafe M, Bunzendahl H, Kraus TW, Büchler MW et al. HEGPOL: randomized, placebo controlled, multicenter, double-blind clinical trial to investigate hepatoprotective effects of glycine in the postoperative phase of liver transplantation [ISRCTN69350312]. BMC surgery 5.1. 2005;5(1):18. 10.1186/1471-2482-5-18 PMC1208918 16105183

[19] Sim Woo-Cheol, Yin Hu-Quan, Choi Ho-Sung, Choi You-Jin, Kwak Hui Chan, Kim Sang-Kyum, Lee Byung-Hoon (2014). L-Serine Supplementation Attenuates Alcoholic Fatty Liver by Enhancing Homocysteine Metabolism in Mice and Rats. The Journal of Nutrition.

[20] Deminice Rafael, de Castro Gabriela S., Brosnan Margaret E., Brosnan John T. (2016). Creatine supplementation as a possible new therapeutic approach for fatty liver disease: early findings. Amino Acids.

[21] Osawa Yosuke, Kanamori Hiromitsu, Seki Ekihiro, Hoshi Masato, Ohtaki Hirofumi, Yasuda Yoichi, Ito Hiroyasu, Suetsugu Atsushi, Nagaki Masahito, Moriwaki Hisataka, Saito Kuniaki, Seishima Mitsuru (2011). l-Tryptophan-mediated Enhancement of Susceptibility to Nonalcoholic Fatty Liver Disease Is Dependent on the Mammalian Target of Rapamycin. Journal of Biological Chemistry.

[22] Tu LN, Showalter MR, Cajka T, Fan S, Pillai VV, Fiehn O et al. Metabolomic characteristics of cholesterol-induced non-obese nonalcoholic fatty liver disease in mice. Scientific reports. 2017;7(1):6120. 10.1038/s41598-017-05040-6 PMC5522413 28733574

[23] Lu Yan, Zhang Jing, Ma Bingqing, Li Kexue, Li Xiaoyu, Bai Hui, Yang Qing, Zhu Xudong, Ben Jingjing, Chen Qi (2012). Glycine attenuates cerebral ischemia/reperfusion injury by inhibiting neuronal apoptosis in mice. Neurochemistry International.

[24] Patro-Malysza Jolanta, Kimber-Trojnar Zaneta, Skorzynska-Dziduszko Katarzyna, Marciniak Beata, Darmochwal-Kolarz Dorota, Bartosiewicz Jacek, Leszczynska-Gorzelak Bozena, Oleszczuk Jan (2014). The Impact of Substance P on the Pathogenesis of Insulin Resistance Leading to Gestational Diabetes. Current Pharmaceutical Biotechnology.

[25] Kiyatake I. Guanidinoacetic acid in serum, urine and renal cortex from streptozotocin-induced diabetic rats. Nihon Jinzo Gakkai shi. 1994;36(6):709–714. 8084071

[26] Wang Dong, Wang Juan, Lu Ming, Song Fei, Cui Qinghua (2010). Inferring the human microRNA functional similarity and functional network based on microRNA-associated diseases. Bioinformatics.

[27] Liu Yang, Li Xueyong, Feng Xiang, Wang Lei (2019). A Novel Neighborhood-Based Computational Model for Potential MiRNA-Disease Association Prediction. Computational and Mathematical Methods in Medicine.

[28] van Laarhoven Twan, Nabuurs Sander B., Marchiori Elena (2011). Gaussian interaction profile kernels for predicting drug–target interaction. Bioinformatics.

[29] Sun Dongdong, Li Ao, Feng Huanqing, Wang Minghui (2016). NTSMDA: prediction of miRNA–disease associations by integrating network topological similarity. Molecular BioSystems.

[30] Vanunu Oron, Magger Oded, Ruppin Eytan, Shlomi Tomer, Sharan Roded (2010). Associating Genes and Protein Complexes with Disease via Network Propagation. PLoS Computational Biology.

[31] Vural Hüseyin, Kaya Mehmet (2018). Prediction of new potential associations between LncRNAs and environmental factors based on KATZ measure. Computers in Biology and Medicine.

[32] Katz Leo (1953). A new status index derived from sociometric analysis. Psychometrika.

[33] Zou Quan, Li Jinjin, Hong Qingqi, Lin Ziyu, Wu Yun, Shi Hua, Ju Ying (2015). Prediction of MicroRNA-Disease Associations Based on Social Network Analysis Methods. BioMed Research International.

